# Editorial: Computational Drug Discovery for Targeting of Protein-Protein Interfaces

**DOI:** 10.3389/fchem.2021.670569

**Published:** 2021-03-19

**Authors:** Massimiliano Meli, Alessandro Pandini, Giulia Morra

**Affiliations:** ^1^Consiglio Nazionale delle Ricerche, Istituto di Scienze e Tecnologie Chimiche Giulio Natta (SCITEC), Milan, Italy; ^2^Computational Biology Group, Department of Computer Science, Brunel University London, Uxbridge, United Kingdom; ^3^Weill Cornell Medicine, Cornell University, New York, NY, United States

**Keywords:** protein-protein interface, drug discovery, theoretical approach, computational methods, protein-protein interaction

Protein-protein and protein-peptide recognition are central in most physiological and pathological cellular processes. Unveiling the molecular basis of these mechanisms of interaction could be the key to understand fundamental biological functions and the underlining causes of complex diseases. In the development of novel therapeutics, targeting specific pathways has become a preferred strategy to overcome issues of specificity and side effects of drugs, but to this end a thorough understanding of the molecular details of PPI is essential. The pandemic emergency brought by the SARS-CoV-2 Virus is the most present-day example of how molecular details on protein-protein interactions and protein binding surfaces are critical to develop treatments for this class of diseases.

Theoretical and computational methods have gained increasing importance in molecular recognition studies. They offer faster cost-effective strategies and can provide directions for experimental design and interpretation of results. For this reason, we believe that the development and application of new theoretical methods to study protein-protein and protein peptide recognition is a timely and exciting topic.

These motivations gave us the cue to write this Research Topic that received a total of 7 contributions: 1 review, 1 perspective, and 5 original researches.

The review of Shechter et al. compares and contrasts conventional and *in silico* High Throughput Screening approaches to identify agents targeting Nuclear Import Inhibitors for Venezuelan Equine Encephalitis Virus Capsid Protein. They conclude that both methods could be a good starting point to enhance the other approach: HTS could give a good starting point for *in silico* drug design process and *in silico* techniques could direct the expensive HTS to more promising leads.

Two novel methods were proposed, one by Santini and Zacharias and one by Iannuzzi et al.. As underlined by Santini and Zacharias, sharp inhibition of pathological PPIs is of significant clinical relevance. The Authors propose a general methodology to quickly develop potential inhibitors by designing cyclic peptides that are able to mimic the epitope, i.e., a portion of the surface responsible for the PPI. They propose an automatic procedure to find the inhibitor within a pre-build cyclic peptide database. The database was built from the analysis of protein-protein complexes present in the Protein Data Bank (www.rcsb.org).

The study of Iannuzzi et al. is focused on a specific protein-protein interaction of essential biomedical relevance, namely the T-cell-antigen interactions. They develop an *in silico* peptide library for the study of *in vitro* or *in vivo* T-cell reactivity (Iannuzzi et al.). The library was built from the docking results of 216 generated peptide against some T cell receptors. In the remaining four original research contributions we can appreciate how computational methods like molecular dynamics, meta-dynamics, and *in silico* screening can be integrated with experiments to find inhibitors of protein-protein interactions or to elucidate the mechanism of binding of a peptide to a protein. As remarked by Yun et al. in their paper the hedgehog (Hh) signaling pathway responsible of embryogenic and tissue homeostasis is overactivated in many cancers. So, the inhibition of this signaling pathway could stop the proliferation of cancer cells. The target along this pathway is Sonic hedgehog (Shh) and in particular its interaction with 12-transmembrane glycoprotein patched (Ptch) protein. The authors were able to identify by virtual screening some inhibitors with an experimental activity on cells, these compounds are a good starting point for further development.

Dailing et al. discuss the inhibition of a ternary protein complex relevant for Osteoarthritis (OA). Post-traumatic arthritis (PTA) involves signaling by the ternary IL-1β/IL-1R1/IL-1RAcP protein and its inhibition could offer a novel approach to the treatment of this disease. In their paper Dailing et al. predict by molecular dynamics simulation and MMGBSA energy calculation that a specific residue on IL-1RAcP could be the key energetic modulator of the ternary complex formation, and prove their hypothesis through monoclonal antibodies targeting it. The peptides built based on this information reveal their high inhibition power.

Chronic inflammation is a process that is associated with many diseases, including cancer proliferation and tumor cell resistance. Leo et al. describe the discovery of a new molecule able to inhibit the formation of a protein complex between the chemokines CXCL12 and the protein High Mobility Group Box 1 (HMGB1) and able to interrupt the cell signaling related to the inflammation process. The drug discovery process started with an *in-silico* evaluation of the ligandability of HMGB1 protein. Then a small molecules database was docked on HMGB1 protein druggable sites and the more promising candidates were selected by clustering. The strongest binder able to disrupt the HMGB1-CXCL12 interaction was confirmed by NMR experiments. Finally, In their paper D'Agostino et al. have addressed the enzyme deoxyhypusine synthase and its inhibitor GC7 with the aim of improving selectivity of the compound, through a comparative study between human DHS (hDHS) and archaeal DHS from crenarchaeon Sulfolobus solfataricus (aDHS). The use of metadynamics to understand the structural and dynamical features of the GC7 inhibition was successful at unveiling the differences and the key residues used in the binding and unbinding process. Experimental validation confirmed these theoretical findings, paving the path to the design of new and more specific inhibitors.

Overall, this collection offers a significant range of approaches and methods covering different aspects of state-of- the-art computational drug discovery, highlighting the importance of integrating diverse computational techniques beyond ligand docking.

## Author Contributions

All authors listed have made a substantial, direct and intellectual contribution to the work, and approved it for publication.

## Conflict of Interest

The authors declare that the research was conducted in the absence of any commercial or financial relationships that could be construed as a potential conflict of interest.

